# A Step Toward Understanding Direct Impacts of a Higher Estrus-Associated Temperature (HEAT): Transcript Level Changes in Cumulus–Oocyte Complexes Directly Exposed to Acute Elevated Temperature

**DOI:** 10.3390/ani15040517

**Published:** 2025-02-12

**Authors:** Jessica L. Klabnik, Jonathan E. Beever, Rebecca R. Payton, Kurt H. Lamour, F. Neal Schrick, J. Lannett Edwards

**Affiliations:** 1 Department of Animal Science, University of Tennessee Institute of Agriculture, Knoxville, TN 37996, USA; jbeever@utk.edu (J.E.B.); rfrazor@utk.edu (R.R.P.); fschrick@utk.edu (F.N.S.); 2 Department of Large Animal Clinical Sciences, University of Tennessee Institute of Agriculture, Knoxville, TN 37996, USA; 3 Department of Entomology and Plant Pathology, University of Tennessee Institute of Agriculture, Knoxville, TN 37996, USA; klamour@utk.edu

**Keywords:** cumulus–oocyte complex, oocyte maturation, estrus, elevated temperature, HEAT, transcripts

## Abstract

Elevated body temperature (HEAT) in sexually receptive females is a normal part of the periovulatory microenvironment. The objective was to identify direct (first 6 h) and delayed (4 h or 18 h of recovery) effects at 41 °C exposure during in vitro maturation (IVM) on transcripts involved in steroidogenesis, oocyte maturation, or previously impacted by elevated temperature using targeted RNA-sequencing. Most transcripts (72.3%) were impacted in the first 2 to 4 hIVM. Twelve of the fifteen transcripts first impacted at 4 hIVM had a higher abundance and three had a lower abundance. Direct exposure to 41 °C impacted the transcripts related to progesterone production and signaling, germinal vesicle breakdown, oocyte meiotic progression, transcriptional activity and/or alternative splicing, cell cycle, cumulus expansion, and/or ovulation. Three transcripts demonstrated a delayed impact; changes were not seen until the COCs recovered for 4 h. The use of multidimensional scaling plots to ‘visualize’ samples highlights that oocytes exposed to an acute elevation in temperature are more advanced at the molecular level during the initial stages of maturation. Described efforts represent important steps towards providing a novel insight into the dynamic physiology of the COC in the estrual female bovid, during HEAT and after body temperature returns to baseline.

## Simple Summary

The body temperature of female cattle increases when they become sexually receptive (i.e., in estrus). Because higher estrus-associated temperatures (HEAT) persist for hours in cattle, direct effects on fertility-important components (i.e., the oocyte and surrounding cumulus cells) are unavoidable and functionally impactful. To test the hypothesis, direct and delayed effects of the physiologically relevant, 41 °C exposure during the early stages of oocyte maturation was evaluated by examining the impact on 47 different targeted transcripts. Most transcripts examined were impacted in the first 2 to 4 h of oocyte maturation. Moreover, the use of multidimensional scaling plots to ‘visualize’ samples provided further evidence that oocytes exposed to an acute elevation in temperature are more advanced at the molecular level during the initial stages of maturation. The consequences and role of the described impacts on the oocyte and its surrounding cumulus cells during maturation are discussed.

## 1. Introduction

Higher estrus-associated temperatures (HEATs) are a hallmark feature in sexually receptive females even when thermoneutral conditions predominate [1]. The maximum level of HEAT typically coincides with the peak of the pivotally important luteinizing hormone (LH) surge [2,3,4], which induces the meiotic resumption and progression of the oocyte resident within the preovulatory follicle (i.e., oocyte maturation) and signifies the end of estrogen dominance, setting the stage for not only the beginning of corpus luteum formation but also ovulation some 26 to 30 h thereafter [5,6]. Although the magnitude and duration of HEAT varies among individual animals, Mills et al. [1] reported that 94% of suckled beef cows at the onset of the breeding season met the clinical definition of hyperthermia (>39.5 °C; [7]). Approximately 49.0% of cows in that study had vaginal temperatures > 40 °C which in some cases persisted up to 6.5 to 10 h, respectively [1]. In some but fewer instances, maximum HEAT exceeded 41.0 °C persisting up to 3.6 h.

When thermoneutral ambient conditions predominate, the level of HEAT and rectal temperature immediately before fixed timed AI have been positively related to fertility. For instance, a higher pregnancy outcome was reported in estrual cows that had rectal temperatures ranging from 38.7 to 40.5 °C immediately before artificial insemination compared to those with rectal temperatures ranging from 37.1 to 38.6 °C (73.5% vs. 60.2%, respectively [8]). When insemination was performed at a fixed time after administration of prostaglandin-F_2α_, a 1 °C increase in rectal temperature at the time of artificial insemination increased pregnancy odds 1.9 times [9].

Although the thermogenic component of estrus is short (average of 15.5 h, ranging from 6.2 to 26.2 h [1]) and ends well before ovulation would be expected [5,6], it is beyond intuitive for ovarian and follicle temperatures to become elevated during this pivotal developmentally important time. The temperature of the reproductive tract increases progressively toward the uterine horns [10], where the ovaries lie in the curvature. In pigs, Hunter et al. [11] showed that in instances where ovarian stromal tissue temperature was elevated during proestrus/estrus, the temperature of mature follicles was also elevated.

Regarding the potential to impact the cumulus–oocyte complex directly, exposure to an acute bout of an elevated temperature in vitro affects meiotic progression. Exposure to 41 °C for as few as 4 h promoted germinal vesicle breakdown (GVBD; [12]). The heat-induced hastening of GVBD compared to thermoneutral controls coincided with decreased gap junction permeability between the oocyte and associated cumulus cells [13]. Furthermore, exposure to an acute physiologically relevant temperature increases the progesterone production by COCs [13,14,15]. Interestingly, progesterone supplementation to the culture medium stimulates bovine GVBD [16] and meiotic progression [17].

Impactful outcomes related to elevated temperatures are not limited to in vitro studies. An acute in vivo bout of hyperthermia occurring after a pharmacologically induced surge of LH and induced by heat stress, resulted in changes in the periovulatory follicular fluid proteome (e.g., proinflammatory-mediator bradykinin, negative acute phase protein-transferrin, interleukin 6 [IL6]) [18]. These findings are intriguing because others have shown the ability of these components to potentiate ovulation, affect oocyte–meiotic progression, and are granulosa and or cumulus cell products [19,20,21,22]. The analysis of the transcriptome, via the bulk RNASeq of the cumulus cells [23] that enveloped oocytes while contained within periovulatory follicles of females exhibiting varying degrees of hyperthermia, identified 25 differentially expressed genes (DEGs) that increased or decreased with each 1 °C change in rectal temperature. Cumulus DEGs involved in cell junctions, plasma membrane rafts, and cell-cycle regulation are consistent with marked changes in the interconnectedness and the function of cumulus after the LH surge. Depending on the extent to which impacts are occurring at the junctional level, temperature-related changes in the cumulus may have indirect but impactful consequences on the oocyte as it resumes and undergoes meiotic maturation [13]. However, findings limited to a single time point (individual COCs were aspirated ~16 h after pharmacological induction of the LH surge [23]) well beyond initial stages of meiotic maturation, preclude further inference.

Mindful of what was known but compelled by what remained unknown about shorter exposure times, the overarching aim of the study described herein was to test the hypothesis that an acute bout of a physiologically relevant elevated temperature directly impacts COC-derived transcripts where corresponding protein product(s) may be involved or promote oocyte maturation, cumulus cell connectivity, expansion, and function (e.g., progesterone production). Heat exposure was limited for up to the first 6 h of in vitro maturation (2, 4, or 6 hIVM). To determine the extent to which heat-related consequences during early maturation may be delayed, transcript abundance in COCs was evaluated later in maturation and after recovery at 38.5 °C for 4 h (10 hIVM) or 18 h (24 hIVM). Examining the potential for the delayed impacts of an elevated temperature is important because these may contribute to the normal microenvironment after HEAT dissipates, which is yet to be described.

Targeted RNAseq was utilized to evaluate the transcript abundance. Doing so provided benefits from the dynamic range associated with next-generation sequencing-based quantification while being limited technically and monetarily by bulk RNAseq. Because targeted RNAseq enables the simultaneous investigation of transcripts, 20 of the cumulus derived transcripts identified by Klabnik et al. [23] that increased or decreased per each 1 °C change in rectal temperature were prioritized. Twenty-seven other transcripts previously shown to be impacted after in vitro heat stress exposure but for longer time periods [13,14,15] and known to be involved in oocyte maturation [14,15,23], cumulus expansion [14,23], and cumulus function [13,14,15,23] were also included. Having the opportunity to do so is of utmost importance because HEAT is an expected part of the normal ovarian preovulatory follicle environment in estrual females. It surprising how little is known about the intrinsic components/factors affected in the cumulus oocyte complex as it undergoes meiotic resumption and progression to metaphase II.

## 2. Materials and Methods

### 2.1. Collection and In Vitro Maturation of Bovine Cumulus–Oocyte Complexes

No animals were used for this work. This study was completed using an estimated 228 abattoir-derived ovaries provided by Southeastern Provision, LLC (Bean Station TN, USA) that followed humane slaughter practices per USDA guidelines and located within 50 min of campus. Prevalent Bos taurus cattle breeds included Angus, Hereford, Charolais and Holsteins. Ovaries were evaluated in pairs upon the removal of the reproductive tract. The presence of a corpus luteum was preferred but not always present. Ovaries with several antral follicles that were not visually atretic (e.g., clear follicle fluid) were used for this study. Reagents and chemicals were obtained from MilliporeSigma (St. Louis, MO, USA) unless otherwise noted. Cumulus–oocyte complexes from abattoir-derived ovaries were collected during January and February and matured as previously described [24]. Oocyte collection and maturation media were prepared as previously described [25].

Ovarian antral follicles 3 to ~12 mm in size were preferentially targeted/slashed using a sterile scalpel blade. The avoidance of larger follicles with larger fluid volumes obviated concerns with collection medium clotting. The selection of COCs having a tight and compacted cumulus with an evenly granulated ooplasm selects against COCs that may have resumed meiosis in larger follicles in response to an endogenous LH surge. Towards this end, 100% of the visually uniform COCs evaluated to date have an intact germinal vesicle (GV [12,26]; 500+ COCs fixed and stained soon after removal from antral follicles). Using maturation conditions previously described [24], >90% of GV-intact COCs would be expected to progress to metaphase II and undergo other important critical maturation events (e.g., cytoplasmic changes in the oocyte and cumulus expansion [12,13,14,26,27]).

**Figure 1:**
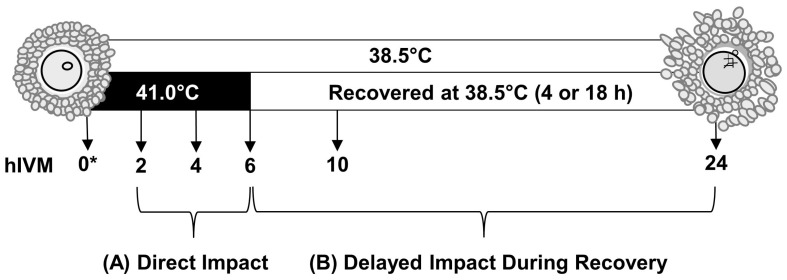
Study schematic. To examine the direct impact of acute elevated temperature occurring in early maturation, subsets of cumulus–oocyte complexes (COCs; n = 35 COCs per pool) were cultured for 2, 4, or 6 h of in vitro maturation (hIVM) at 41 °C. To examine how COCs recover from an acute exposure to elevated temperature occurring during early maturation (i.e., delayed impact), COCs were cultured at 41 °C for 6 hIVM and then allowed to recover at 38.5 °C for an additional 4 h (10 hIVM total) or 18 h (24 hIVM total). Subsets of COCs were cultured for 2, 4, 6, 10, or 24 hIVM at 38.5 °C for comparison. * Whenever sufficient numbers were present, a subset of COCs was also processed soon after removal from ovary to provide a 0 hIVM group.

Cumulus–oocyte complexes with compact cumulus vestments and homogenous ooplasm underwent in vitro maturation in polystyrene tubes; Sarstedt AG and Co. Nümbrecht, Germany). Per each oocyte collection day (i.e., experimental replicate), COCs (n = 35 per 0.5 mL maturation medium; experimental unit) were cultured at 38.5 °C (thermoneutral) for 2, 4, 6, 10, or 24 hIVM (Figure 1). To examine the direct impact of a physiologically relevant elevated temperature, COCs were exposed to 41 °C for 2, 4, or 6 hIVM. To examine the possible delayed impacts, COCs were cultured at 41 °C for 6 hIVM and then transferred to 38.5 °C for an additional 4 h (10 hIVM total) or 18 h (24 hIVM total) of recovery. This ultimately resulted in a 2 × 5 factorial treatment arrangement. Whenever sufficient numbers were available (5 of the 6 days of COC collection), a subset of COCs was also processed soon after removal from antral follicles to provide a 0 hIVM group. Incubator temperatures were validated using internal mercury thermometers.

Per each hIVM, COCs were removed from culture, washed in HEPES-TL containing 0.1% polyvinyl alcohol before lysis in extraction buffer (Quick-RNA Kit; Zymo Research, Irvine, CA, USA). Lysates were stored at −80 °C until RNA isolation [15]. This study was replicated on six different occasions and utilized a total of 2275 COCs.

### 2.2. Total RNA Isolation and cDNA Synthesis

Total RNA was isolated using Quick-RNA Microprep kits (Zymo Research) as per the manufacturer’s instructions. DNAse treatment was performed on column with TURBO DNase (Thermo Fisher Scientific, Waltham, MA, USA). The concentration of RNA was determined using the Qubit RNA High Sensitivity assay (Invitrogen/Thermo Fisher Scientific, Waltham, MA, USA). Two COC pools were removed due to the low concentration of isolated RNA: one that was cultured at 38.5 °C for 4 hIVM and one that was processed at 0 hIVM. Isolated RNA was analyzed using RNA 6000 Nano kit on an Agilent 2100 Bioanalyzer or a standard RNA Screen on an Agilent 4200 TapeStation (Agilent Technologies, Santa Clara, CA, USA). RNA integrity number values ranged from 4.6 to 9.7 (mean ± standard deviation: 8.7 ± 0.1). Isolated total RNA (125 ng per pool of COCs) was converted to cDNA using the High Capacity cDNA Reverse Transcription kit (Thermo Fisher Scientific) with oligo(dT)_18_ primers (Thermo Fisher Scientific) before storage at −80 °C.

### 2.3. Primer Design, Library Preparation, and Targeted Messenger RNA-Sequencing

Primers were designed using approximately 200 bp of sequence from the *Bos taurus* genome (Reference ARS-UCD1.2 Primary Assembly) with BatchPrimer3 to produce amplicons of 59 to 79 bp in length ([28]; Table 1). Only primer sets confirmed to span the exon–exon junction were utilized. Library preparation was performed on study cDNA in triplicate and sequenced using a Hi-Plex approach [29,30]. Targeted RNA sequencing was performed on an Illumina MiSeq device using a 2 × 150 configuration [31] by Floodlight Genomics (Knoxville, TN, USA). Raw sequence reads were trimmed of identifiers using CLC Genomics Workbench version 9.5.3. The quality of the reads was assessed using FastQC software, Version 0.11.9 [32]. Trimming was performed with SeqPurge [33] and Trimmomatic [34]. The per sequence quality score measured in the Phred quality scale was above 25 on average for all the samples [32]. Reads were then aligned to the *Bos taurus* transcriptome (ARS-UCD1.2) and counted using Salmon [35]. The trimmed means of the M values method of data normalization was performed using the EdgeR package [36,37,38], resulting in counts per million (CPM). Transcripts were retained for further analysis only if there was >2200 CPM in a minimum of 24 of the technical triplicates. Raw counts were then normalized in EdgeR and RUVr (k = 4) as part of the RUVSeq package [39]. Multidimensional scaling (MDS) plots of CPM reflecting either impacts of acute exposure to 41 °C in early maturation or subsequent recovery were generated with the Glimma package [40]. In the MDS plots, technical triplicates clustered closely together; therefore, the CPM of the technical triplicates for each transcript in each sample were averaged prior to statistical analyses.

### 2.4. Data and Statistical Analyses

In order to test hypotheses that each transcript may be directly impacted by an acute exposure to 41 °C in early maturation and may or may not recover later during later maturation, abundance (counts per million) of each transcript was analyzed individually as a randomized block design using generalized linear mixed models (PROC GLIMMIX, SAS 9.4, SAS Institute, Cary, NC, USA), blocking on the day of COC collection. Fixed effects per each transcript model included IVM temperature (38.5 and 41.0 °C), hIVM, and the respective interaction (IVM temperature × hIVM). Using this approach allowed for alpha (i.e., Type I Error Rate) to be set at 0.05 [41]. The only transcript data that were not normally distributed per Shapiro Wilk’s was CAV1, which had a unique expression pattern related to hIVM, and shown in results. Therefore, CAV1 data were also analyzed as a 2 × 4 factorial treatment arrangement with eight treatment combinations (2, 4, 6, and 10 hIVM); in this arrangement, data were normal (W = 0.91). Per each transcript, treatment combination differences were determined using Fishers protected least significant differences [42] and are reported as least squares means ± standard error. Average abundance (counts per million) for each transcript in subsets of COCs evaluated at 0 hIVM are provided for visual comparison but were not included in statistical analyses.

## 3. Results

**Figure 2:**
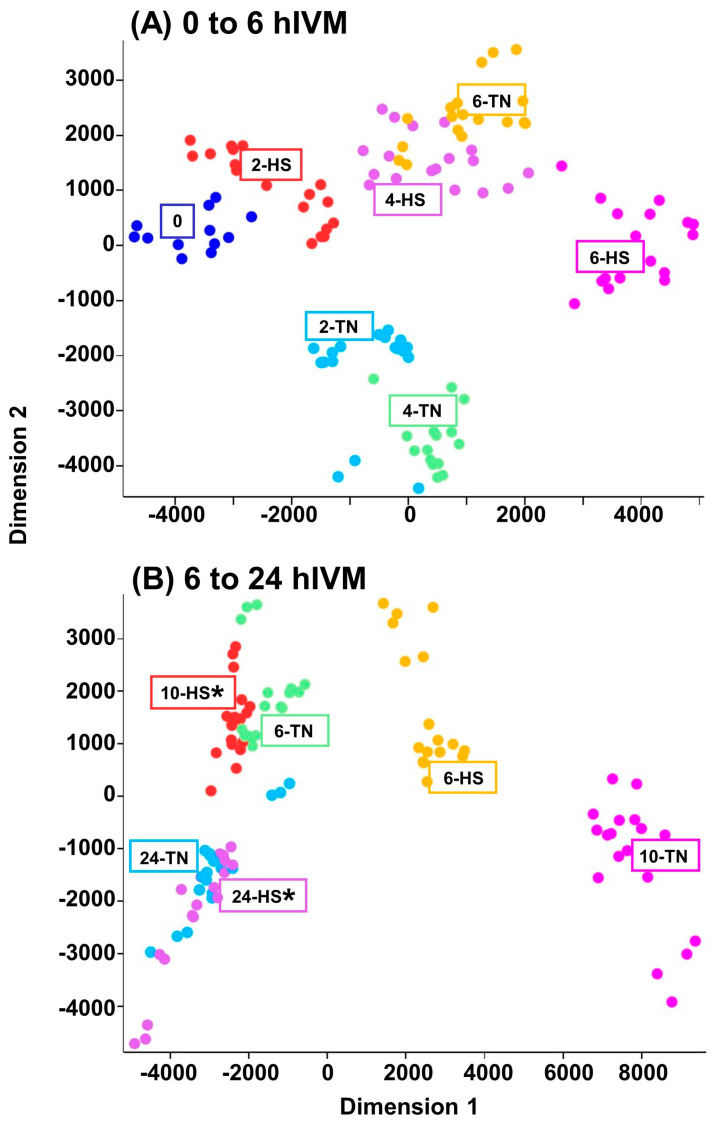
Multidimensional scaling plots highlighting differences in counts per million that resulted from efforts to examine abundance of 47 different targeted transcripts in cumulus–oocyte complexes. (**A**) COCs were cultured at 38.5 (Thermoneutral-TN) or 41 °C (Heat Shock-HS) for the first 2, 4 or 6 hIVM; 0 hIVM were included as a reference; (**B**) COCs were matured at 41 °C for 6 hIVM (6-HS) or allowed to recover at 38.5 °C for 4 h (10-HS*) or 18 h (24-HS*), thereafter in comparison to COCs cultured at 38.5 °C for 6, 10, and 24 hIVM (TN).

Multidimensional scaling plots representing each in vitro maturation time and temperature treatment combination and performed in triplicate are provided in Figure 2. Differences in clustering related to time and temperature in early maturation were apparent. Interestingly, and relevant for the first 6 hIVM, samples originating from COCs directly exposed to 41 °C for 4 hIVM overlapped with those matured at 38.5 °C for 6 hIVM (Figure 2A). When allowed to recover at 38.5 °C for 4 h, samples originating from COCs directly exposed to 41 °C for 6 hIVM (10-HS in Figure 2) began to overlap with those matured at 38.5 °C for 6 hIVM (Figure 2B). Samples from COCs directly exposed to 6 h of 41 °C but not allowed any recovery time clustered between those originating from COCs matured at 38.5 °C for 6 and 10 hIVM. By 24 hIVM, however, samples from COCs matured at 38.5 °C (24-TN in Figure 2) versus those that were directly exposed to 41.0 °C for the first 6 hIVM but allowed to recover for a total of 18 h at 38.5 °C (24-HS in Figure 2) clustered with the most overlap (Figure 2B).

**Figure 3:**
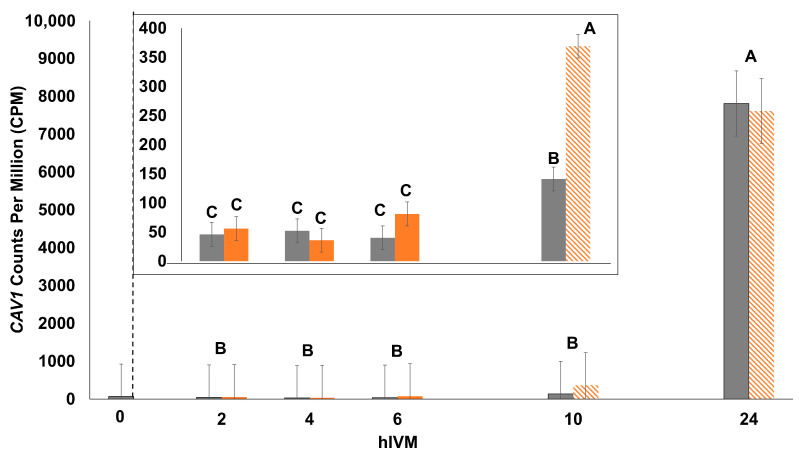
Impact of 41.0 °C exposure during the first 6 hIVM on *CAV1* count per million (CPM) of cumulus–oocyte complex (COC). COCs were cultured at 41.0 °C for 2, 4, or 6 hIVM (orange bars), or allowed to recover at 38.5 °C for an additional 4 h (10 hIVM) or 18 h (24 hIVM; hashed bars as indicated by 41 °C). Thermoneutral COCs were cultured at 38.5 °C for 2, 4, 6, 10, or 24 hIVM (gray bars). Bars (least squares means ± SEM) with different letters differ significantly for hIVM. Temperature and the interaction of maturation temperature and hIVM was not significant. The inset reflects differences when data were analyzed with only the 2, 4, 6, and 10 hIVM timepoints. Bars with different letters (i.e., A through C) through differ significantly for the interaction of maturation temperature and hIVM. COCs at 0 hIVM were included as a reference.

The abundance of the majority of individual transcripts (43 out of 47 total examined) differed depending on hIVM and IVM temperature (hIVM * IVM temperature interaction; *p* ≤ 0.05; Table 2, Table 3 and Table 4). The first hIVM in which IVM temperature impacted transcripts with described roles in progesterone production and/or signaling, cellular signaling, the β-catenin complex, the cell cycle, or cumulus expansion is provided in Table 2. Table 3 includes the first hIVM where IVM temperature impacted abundance of Src Family Kinase transcripts. Whereas Table 4 lists the first hIVM where IVM temperature impacted the abundance of transcripts with less defined roles in the COC but they, or their gene products, were reported by others to have been impacted by heat in cumulus cells, the oocyte, and/or in the follicular fluid. Due to the range of counts per million, *CAV1* data were not normally distributed when analyzed as a 2 × 5 factorial treatment arrangement and only hIVM was significant (*p* < 0.0001; Figure 3). When only the earlier hIVM were included in the model (i.e., 2, 4, 6, and 10 hIVM), data were normal and the interaction was significant (hIVM * IVM temperature *p* < 0.0001; Figure 3 Inset).

Interestingly, 19 out of the 43 transcripts examined (44.1%) were impacted by 41 °C exposure after only 2 hIVM (6 were of higher abundance versus 13 of lower abundance; Table 5). Of the 15 transcripts with a first noticeable impact at 4 hIVM, 12 were of higher abundance whereas 3 were of lower abundance. Altogether, 34 (79.1%) and 40 (93.0%) out of 43 transcripts were first noticeably impacted by 4 and by 6 hIVM, respectively.

Pertaining to the four other transcripts examined (*TFRC*, *CCDC80*, *SNAP91*, and *RAI14*); *TFRC* was impacted by the IVM temperature (*p* = 0.004) and hIVM (*p* = 0.007). The only impact on *CCDC80* was hIVM (*p* < 0.0001) but not IVM temperature (*p* = 0.5). Similarly, *SNAP91* was impacted by hIVM (*p* = 0.0003) but not IVM temperature (*p* = 0.5). Although *RAI14* was present (24,958 to 28,129 CPM), it was not impacted by hIVM (*p* = 0.4) or IVM temperature (*p* = 0.9).

## 4. Discussion

An overarching aim of the novel study described herein was to test the hypothesis that a short, acute bout of a physiologically relevant elevated temperature directly impacts COC-derived transcripts where corresponding protein product(s) may be involved in or affecting cumulus cell connectivity, expansion, and or function (e.g., progesterone production, cell signaling, cell cycle, β-catenin complex or other) which may indirectly or directly promote oocyte maturation. Allowing time for COCs to recover after heat exposure provided the additional opportunity to identify changes, if any, that may contribute to the normal microenvironment in the cow after HEAT dissipates. Deliberate effort to do this study during winter and abruptly expose naïve COCs to short bouts of heat “shock” in vitro, while not perfect, is necessary to gain new knowledge about possible intrinsic physiological events that may be occurring at the level of the cumulus to mediate meiotic resumption and progression of the oocyte, especially in circumstances where developmental outcomes are expected to be favorable.

When COCs were exposed to a short bout of heat in our study, the surrounding cumulus cells were intimately associated with the oocyte, projecting through the zona pellucida and into the oolemma via gap junctions [43]. Intimate connections with the oocyte permit bidirectional exchange of small molecules [44]. Cumulus presence is essential for meiotic resumption and the developmental progression thereafter [45,46]. Even after gap junctional connections breakdown, cumulus cells continue to envelop the oocyte providing critical support [47]. Thus, COCs remained intact before lysis.

Collectively, novel results described herein highlight direct and delayed effects on transcript abundance after short bouts of elevated temperature (i.e., 2, 4, or 6 h) were applied during the beginnings of oocyte maturation (i.e., meiotic resumption through GVBD). Of the 47 transcripts examined, 44 were impacted by heat with direct impacts noticed as early as 2 hIVM (majority of transcripts were affected after a 4 h exposure). Specific to impacts on the 20 in vivo-derived cumulus transcripts identified by Klabnik et al. [23] where abundance increased or decreased per each 1 °C change in rectal temperature of hyperthermic cows, 17 whose protein products are known to be involved in cell junctions, plasma membrane rafts, and cell-cycle regulation and other functions were directly impacted by heat as early as 2 and 4 hIVM.

Cumulus cells are transcriptionally active [48] and likely contribute much of the heat-related findings described herein. Despite having few to no LH receptors initially, cumulus begins to produce progesterone as oocyte maturation progresses [49,50]. In contrast, the transcriptionally quiescent oocyte relies on a depleting pool of maternal RNA and proteins after meiotic resumption [51,52]. Oligo-dT primers were used in our study to ascertain changes in transcript abundance likely to have a functional consequence [53]. The abundance patterns of certain transcripts (e.g., MMP9, CAV1, and IL6R) in COCs in our study were highly repeatable with patterns previously observed in COCs or cumulus cells alone after exposure for 12 h to 41 °C [14,15].

Interestingly, many of the targeted transcripts affected by heat exposure after 2 h were lower in abundance, whereas those affected by heat after 4 h were mostly higher in abundance. To gain a better perspective of these outcomes, COC samples were ‘visualized’ using multidimensional scaling. Remarkably, the targeted ‘transcriptome’ in COCs exposed to 41 °C for 4 h was more like the targeted transcriptome in COCs matured at 38.5 °C for 6 hIVM. The targeted transcriptome in COCs exposed to 41 °C for 6 h was in between that of COCs matured for 6 and 10 h at 38.5 °C. This finding is the first we are aware of to document a heat-induced shift at the transcriptomic level in the COC when considering numerous transcripts simultaneously within the same sample. Although the functional consequences of this molecular signature remain unclear, it is remarkable that the timing of the heat-induced shift in the targeted transcriptome overlaps/fits well with previous observations showing that the direct exposure of COCs to 41 °C for as few as 4 h promoted earlier germinal vesicle breakdown (GVBD; i.e., heat shocked oocytes underwent GVBD sooner and mature faster than thermoneutral controls; [12]). The follow-on efforts of Campen et al. [13] confirmed that the heat-induced hastening of GVBD in vitro coincided with decreased gap junction permeability between the oocyte and associated cumulus cells. Relevant for meiotic progression and subsequent development, in vitro exposure to 41 °C for up to 6 hIVM directly promotes GVBD [12,13,26] without negative impacts on meiotic progression to metaphase II [12,26] or early embryo development after fertilization [15,54,55]. Mindful of this, it is unsurprising that the 24 hIVM samples (COCs exposed to 38.5 °C and 41 °C for the first 6 hIVM but recovered for 18 h) overlapped on the multidimensional scaling plot. Depending on the extent to which HEAT exposure in vivo may be shifting developmentally important processes, additional effort may be needed to investigate insemination timing in circumstances where body temperature elevations are more pronounced and persist for a longer time.

A member of the Src-family kinases, FYN, was differentially expressed in the in vivo-derived cumulus transcripts identified by Klabnik et al. [23] in cows exhibiting varying levels of hyperthermia. This finding was especially intriguing because members of the Src-family have a large degree of functional overlap [56] and may impact meiotic maturation. Follow-on efforts in our study identified four Src-family kinases that were directly impacted by exposure to 41 °C (SRC, FYN, LCK, and FGR). Greater abundance in SRC at 2 hIVM and LCK at 4 hIVM were particularly notable. Although the significance of these findings remain unclear, others have shown that non-specific Src-family kinase inhibitors reduce GVBD (rats: [57]; mice: [57,58]; porcine: [59]). Src induces GVBD by binding to the progesterone receptor in Xenopus oocytes [60], whereas FYN has been shown to promote GVBD in mice [61].

Progesterone production by the cumulus cells increases as in vitro oocyte maturation progresses [13,14] and levels are higher after heat exposure [13,14,62]. Heat-induced increases in progesterone production may be related to changes in transcript abundance [13,14,15]. Interestingly, samples exposed to heat had a lower abundance of FDX1 and FDXR but a higher abundance of HSD3B7 at 2 and 4 hIVM. FDX1 and FDXR encode enzymes that donate electrons, perpetuating catalytic activity, to cytochrome p450 enzymes such as p450scc, which convert cholesterol to pregnenolone in the process of steroidogenesis. Pregnenolone is converted to progesterone by 3-beta-hydroxysteroid dehydrogenase (3βHSD, [63]).

The consequences of exposure to elevated temperature on progesterone signaling remain unclear. Genomic pathways of progesterone signaling rely upon HSP90 protein chaperones [64] in which progesterone binds to its nuclear receptors (PRα and PRβ) to regulate the transcription [65]. Herein, heat exposure altered HSP90AB1 and HSP90B1 transcript abundance throughout maturation which may impact progesterone signaling via traditional pathways. It was recently reported that heifers with a negative fertility breeding value had an increased expression of HSP90B1 in COCs collected near estrus [66]. However, progesterone signaling may also occur through non-genomic, membrane-bound progesterone receptors (PGRMC1 and PGRMC2; [65]); PGRMC2 transcript levels were impacted throughout oocyte maturation by heat beginning at 2 hIVM. Collectively, results suggest a role of progesterone and its downstream events in changes to COC physiology observed during IVM and in vivo maturation after short-term heat exposure.

It is possible that MMP9 was impacted by increased progesterone production by COCs exposed to physiologically relevant elevated temperature [13,14,15] as transcript abundance was lower in recovering COCs despite an initial higher abundance at 4 hIVM at 41 °C. Progesterone was inversely related to MMP9 levels in COCs exposed to 41 °C for 12 h [62] and suppressed MMP9 secretion in other cell types (cervical fibroblasts: [67]; placental cells: [68]). Rispoli et al. [14] reported similar findings regarding COCs that recovered from a 12 h exposure to 41 °C in early maturation. Decreased MMP9 coincided with a decreased secretion of the proMMP9 enzyme, which negatively impacted embryo development in bovines [14] and humans [69]. However, developmental competence was not rescued by the supplementation of the human proMMP9 [62].

Interleukin 6 (IL6) is promoted by progesterone production and may regulate COC function, cumulus expansion, and oocyte developmental competence [21]. In the current study, IL6R decreased over time, consistent with the outcomes from Rowinski et al. [15]. The binding of IL6 to its receptor can induce the JAK-STAT pathways [21] and CCDC80 increases the phosphorylation of STAT3 [70]. Interestingly, CCDC80 had a similar pattern of expression to IL6R (i.e., decreased through the first 10 hIVM), despite not being affected by temperature. The decline in CCDC80 may be functionally important as CCDC80 is positively regulated by estrogen in the uterus and mammary gland [71], estrogen declines in the follicle after the LH surge [72], and the addition of estrogen during in vitro maturation inhibited bovine oocyte nuclear maturation [73].

Another pathway that can be induced by IL6 is ERK1/2 signaling [21], which may be involved in gap junction communication and progesterone production [74,75,76]. The proportion of active ERK1/2 in cumulus cells increased in early maturation [13]. This was not reflected in the levels of MAPK3 transcripts (i.e., the gene encoding ERK2) within COCs in the current study, which were relatively stable during early maturation with heat-induced reductions at 6 hIVM. Alternatively, AMPK may also affect gap junction communication through the control of protein synthesis and progesterone production [50,77]; the gene encoding AMPK (PRKAA1) had a lower abundance under the exposure to 41 °C at 6 hIVM in the current study. Campen et al. [13] reported that AMPK peaked at 6 hIVM in COCs under thermoneutral conditions and culture at 41 °C tended to a lower active AMPK. This further supports the notion that a decline in AMPK, which may have a role in accelerated meiotic progression [50], could be due to elevated temperature.

The upregulation of TNIK in samples exposed to 41 °C for 4 h in the current study coincides with the timing of the earlier expression of IL6 and its signal transducer [15]. Others have reported that IL6 promoted the interaction of the TNIK/β-catenin/TCF4 complex (multiple myeloma cells: [78]); transcriptional activity of the β-catenin/TCF4 complex is promoted by TNIK [79] and inhibited by SF1, which induces an alternative splicing activity [80]. The timing of TNIK’s higher abundance coincides with a lower level of SF1, suggesting a shift towards earlier transcription. The abundance of SF1 transcripts was higher at 6 h in samples exposed to 41 °C, which suggests changes in alternative splicing during maturation. Furthermore, the estrogen receptor ESR2 may be alternatively spliced by β-catenin to an isoform with dominant negative activity (HeLa cells: [81]). In the current study, ESR2 was present throughout maturation and was of higher abundance when exposed to 41 °C at various hIVM. Prior to the LH surge, estrogen signaling maintains the oocyte in meiotic arrest [82] and the addition of estrogen during in vitro maturation inhibited bovine oocyte nuclear maturation [73]. Altogether, it is possible that that exposure to elevated temperature increases progesterone production [13,15] to promote/accelerate IL6 production [15,21], resulting in altered regulation β-catenin/TCF4 complex activity (through TNIK and SF1) to stimulate transcription and subsequently the alternative splicing of ESR2. The potential alternative splicing of ESR2 to a dominant negative form could possibly then allow for the nuclear maturation of the oocyte, despite the presence of the receptor mRNA throughout maturation.

The unique cell type of the cumulus versus the oocyte when analyzed as a complex creates difficulty in concluding the impacts of exposure to 41 °C during maturation on cell cycle regulation. Transcripts that are generally thought to have a role in cell cycle regulation may be of importance because, while the oocyte is undergoing nuclear maturation (but not DNA replication), the cumulus cells may no longer be mitotically active [83]. The cumulus cell MDM2-p53-NR5A1 pathway was previously described to impact oocyte quality, ovulation, and fertilization [84], where the increased expression of MDM2 and NR5A1 were correlated with positive outcomes. In the current study, MDM2 had lower levels of abundance when exposed to directly elevated temperature at 2 and 4 hIVM which could possibly relate to higher levels of BANP, a stabilizer of p53 [85], at 4 and 6 hIVM. While this could indicate a negative impact on the oocyte, NR5A1 had higher abundance when exposed to 41 °C for the majority of pairwise comparisons, suggesting the opposite. Furthermore, TP53 was only impacted by elevated temperature at 4 hIVM. In contrast, bovine cumulus cell BANP expression was decreased in response to a 12 h heat exposure at 16 h maturation in vivo [23] and at 24 h maturation in vitro [14]. Differences may be attributed to the fact that RNA was extracted from the entire COC in the current study, compared to only cumulus cells in Haraguchi et al. [84], Klabnik et al. [23], and Rispoli et al. [14]. Lower levels of PAK2 when exposed to 41 °C at 2 hIVM in the current study could potentially contribute to the heat-induced hastening of GVBD and progesterone production [13]. PAK2 maintains Xenopus oocyte meiotic arrest [86] and inhibits progesterone production in human granulosa cells [87]. The elevated levels of ARHGAP6 and ARHGAP31 and lower levels of MCM6 in early maturation when exposed to 41 °C may indicate the cessation of cell cyclicity. ARHGAPs inactivate GTPases to halt mitosis and cytoskeletal rearrangement [88]. The helicase complex that contains MCM6 is critical to DNA replication [89]. The complexity of the relationship between cumulus and oocyte as maturation events occur requires further study, independently of the effect of heat.

Three transcripts analyzed in the current study have been reported to positively impact cumulus expansion: FSHR, INHBB, and PRDX2. At the majority of hIVM in the current study, the abundance of FSHR was lower in COCs exposed to 41 °C but INHBB had a higher abundance. Cumulus expansion in bovine oocytes was induced by FSH and higher levels of FSHR were present in better quality COCs [90]. INHBB enabled FSH-induced cumulus cell expansion (mouse: [91]) and its expression increased from GV to MII stage in bovine oocytes [92]. In the current study, when exposed to 41 °C, PRDX2 had a lower abundance at 2 hIVM and after 4 h of recovery (10 hIVM), but higher abundance at 6 hIVM. A cumulus expansion was promoted by PRDX2 in mice [93]. Previously, an acute hyperthermia decreased PRDX2 in bovine cumulus cells ~16 h after a pharmacologically induced LH surge [23]. The abundance of the sulfa-transporter SLC26A2 was higher after exposure to 41 °C in the current study and after acute hyperthermia in bovine cumulus cells [23]. Although a direct role in the COC is unknown, sulfonated proteoglycans are thought to be required for successful COC expansion [94]. While prolonged heat shock reduced cumulus expansion [95], effects of shorter durations of elevated temperature (positive or negative) have not been noted by our laboratory (Edwards, unpublished observation).

Since our study focused on early maturation, it is unsurprising that the majority of targeted transcripts were impacted in the first 6 h. Only three transcripts demonstrated a delayed impact (i.e., first affected during recovery from heat) from exposure to 41 °C in early maturation (LYN, YES1, and CAV1) with differences first noted at 10 hIVM. Our lab previously reported that the elevated temperature decreased CAV1 at 24 hIVM but not at 12 hIVM [14]. Differences may be due to the shorter length of exposure to 41 °C (i.e., 6 h compared to 12 h) or the presence of oocytes (i.e., COC versus cumulus only) in the current study. Nevertheless, both studies demonstrated a similar pattern of expression over time. Although CAV1 precipitates with FSHR [96], its delayed expression suggests that it is not crucial for FSHR signaling in early maturation. The drastic upregulation late in maturation appears to be quite unique and is intriguing, particularly as the role of CAV1 in late maturation or events occurring after ovulation is unknown. Of the 43 transcripts that had a significant temperature by time interaction, only one transcript remained affected by 18 h of recovery (MMP9).

## 5. Conclusions

Because higher estrus-associated temperatures (HEATs) persist for hours in cattle, direct effects on fertility-important components (i.e., the oocyte and surrounding cumulus cells) are unavoidable and likely functionally impactful. Building off a foundation of knowledge, novel findings described herein highlight both the direct and delayed effects of short bouts of a physiologically relevant elevated temperature (i.e., 2, 4, or 6 h) when applied during the beginnings of oocyte maturation (i.e., meiotic resumption through GVBD). Direct consequences on COCs were noted as early as 2 hIVM and included impacts on transcript abundance whose protein products are known to be involved in cell junctions, plasma membrane rafts, and cell-cycle regulation and other functions that may indirectly or directly impact meiotic resumption and progression. Novel findings documenting a heat-induced shift in targeted transcriptome provide additional support for the heat-shocked COC to be more advanced. Depending on the extent to which HEAT exposure in vivo may be shifting developmentally important processes, additional effort may be needed to investigate insemination timing under circumstances where body temperature elevations are more pronounced and persist for a longer time.

## Figures and Tables

**Table 1:** Official gene symbols and nomenclature of the primer sequences used for targeted RNA sequencing.

Official Gene Symbol	Gene Name	GenBank Accession Number	Amplicon Length (bp)	Forward Primer Sequence	Reverse Primer Sequence
*ARHGAP31*	Rho GTPase activating protein 31	NM_001205810.1	62	CGGGAGTGTATTTGTGAGAGG	TTAGCTGGTCGGATGGTAGC
*ARHGAP6*	Rho GTPase activating protein 6	NM_001191354.1	69	TGGTGCAGAAAATGATCGAA	AGCACTTCATTCTGCAGATCC
*BANP*	BTG3 associated nuclear protein	NM_001075620.1	67	CAGGTCCAGATCCACCAGA	ACCTGGGCGATGTGTAGG
*BDKRB1*	Bradykinin receptor B1	NM_001109999.1	74	TCCCTTTCATTTTGCCTGAG	GGCTCTGGTTGAGAGTCTGG
*CAV1*	Caveolin 1	NM_174004.3	79	GTCTTCAACCAGCCACGAG	CGGATGGGAACAGTGTAGAGA
*CCDC25*	Coiled-coil domain containing 25	XM_015472401.2	72	CGGCTCATGTGTACCTTCG	CAGTCCATCAGCACCTCCTT
*CCDC80*	Coiled-coil domain containing 80	NM_001098982.2	67	CAGCTCTCTGCCCTCAGTG	AAAAGCTTCAGAACCGTTATGTG
*CTSB*	Cathepsin B	NM_174031.2	78	CTACAAAAATGGCCCAGTCG	GTGCTGGTACACCCCAGACT
*ESR2*	Estrogen receptor 2	NM_174051.3	59	GGACAGGGATGAAGGGAAAT	GCCAGGAGCATGTCAAAGAT
*ETFA*	Electron transfer flavoprotein subunit α	NM_001075822.1	65	CAGCATTTAGCTGGGATGAA	TGGAGCTTCTGGGTCTTTGT
*FDX1*	Ferredoxin 1	NM_181011.2	66	TTGATGGTTTTGGTGCATGT	TGCTGTTCAAAGATGAGGTGA
*FDXR*	Ferredoxin reductase	NM_174691.1	68	GATGTGCCAGGCCTCTACTG	CATGGTGGTGGTGATGACA
*FGR*	FGR proto-oncogene, Src family tyrosine kinase	NM_001098991.1	76	GGAGGGTCATGTTTTGAAGC	GCTCCATGTAGGCCATGC
*FSHR*	Follicle stimulating hormone receptor	NM_174061.1	60	ATGTTTTCCAGGGAGCCTCT	GAACGGATCCTGGTTCTTGA
*FYN*	FYN proto-oncogene, Src family tyrosine kinase	NM_001077972.1	63	CCAGGTTGATCGAGGACAAT	TCCACTTGATGGGGAACTTG
*HSD3B7*	Hydroxy-delta-5-steroid dehydrogenase, 3-beta- and steroid delta-isomerase 7	NM_001034696.1	68	CTAGCTGAGCAGCTCGTCCT	ACATGTCACTAGGGGCAACC
*HSP90AB1*	Heat shock protein 90 α family class B member 1	NM_001079637.1	69	AAGAAATTCTATGAGGCGTTCTC	GTCGCCGGTTAGTGGAGTC
*HSP90B1*	Heat shock protein 90 β family member 1	NM_174700.2	77	CACCCACTAATCAGAGACATGC	AACCACAGCAAGATCTGAAACA
*HTATIP2*	HIV-1 Tat interactive protein 2	NM_001040563.1	71	GGGAGCTGATAAGTCAAGCAA	AACTCTTCAACTCTAGCTTCCA
*IL6R*	Interleukin 6 receptor	NM_001110785.3	62	TCCAAAGATTCTGCAAAAACAA	GGGCAGTGGTACCGAAGTAG
*INHBB*	Inhibin subunit β B	NM_176852.2	79	CGTCTCCGAGATCATCAGC	CGTTGGAGATGAAGAAGTACAGG
*LCK*	LCK proto-oncogene, Src family tyrosine kinase	NM_001034334.1	75	TTGGTCTAGCACGCCTCATT	GCTGTCCACTTAATGGGAAACT
*LYN*	LYN proto-oncogene, Src family tyrosine kinase	NM_001177740.2	67	AGGAGCCCATCTACATCATCA	ATCGCTCTTCAGGAAATCCA
*MAPK3*	Mitogen-activated protein kinase 3	NM_001110018.1	69	CACCCCTACCTGGAGCAGTA	TGTCGAAGGTGAAAGGTTCC
*MCM6*	Minichromosome maintenance complex component 6	NM_001046234.1	65	CGTCTCTCTGAAGCGATGG	TTCCTTCACATGTTTAGGCTGA
*MDM2*	MDM2 proto-oncogene	NM_001099107.1	73	CCTCAGCGAAGAAGGACAAG	CGCCTGCCTGATACACAGTA
*MMP9*	Matrix metallopeptidase 9	NM_174744.2	59	GAGGGTAAGGTGCTGCTGTT	CTGTGTCTTCACGTCGAACC
*NR5A1*	Nuclear receptor subfamily 5 group A member 1	NM_174403.2	70	TACCGTCAGATTCAGCATGG	GTGGTCAGCTCCACCTCCT
*PAK2*	p21 (RAC1) activated kinase 2	XM_024984244.1	70	GGAGAGCCTCCATACCTCAA	TCTGGGGTTCCATTAGTTGC
*PEAK1*	Pseudopodium enriched atypical kinase 1	XM_024982035.1	69	CCTGAATCCCTGTAGTGCAA	ATGCTTGGGAGGTATCATGG
*PGRMC2*	Progesterone receptor membrane component 2	NM_001099060.1	66	TGTTCGAGAATGGGAAATGC	CCCTGGTTTTAGGAGTCTGC
*PRDX2*	Peroxiredoxin 2	NM_174763.2	62	ATTATGGCGTGCTGAAGGAA	TGCCGTCGATGACAAAGAG
*PRKAA1*	Protein kinase AMP-activated catalytic subunit alpha 1	NM_001109802.2	60	TTGTGGCTCACCCAACTATG	TGGACCAGCATACAATCTTCC
*RAI14*	Retinoic acid induced 14	XM_005221621.4	62	CAGCAGCAGGTCAAACAGC	CACTTCCTGGTGTTGTTTCTTG
*RBM3*	RNA binding motif protein 3	NM_001303463.1	64	TGGATATGGAAGGTCCAGAGA	CCCCTGAGTAGCGGTCATAA
*SCN7A*	Sodium voltage-gated channel alpha subunit 7	XM_010802033.3	77	TGCCAATTTCCACAAGCATA	CGATACTGTTTCTTCTGTTTCACTG
*SF1*	Splicing factor 1	NM_001081614.1	65	TACCTGGGAAGTACGCCTGT	CGGCATCATACCTTTTCCTT
*SLC26A2*	Solute carrier family 26 member 2	NM_001040525.1	61	TGGCAGCACTGTAACCTTTG	CCACTTGAAAGAAGCCCATT
*SNAP91*	Synaptosome associated protein 91	NM_001105378.1	70	GAGGCCACCCTTTGGAGT	TCTGAGAGGCAGGTGTAGGG
*SNX22*	Sorting nexin 22	XM_005211652.4	61	AGGGCTTGGAGGCCTATATC	TAGTTCCTTGGGCACATCCT
*SRC*	SRC proto-oncogene, non-receptor tyrosine kinase	NM_001110804.2	69	CTGGCTCGACTCATTGAAGA	CTGTCCACTTGATGGGGAAT
*TANC1*	Tetratricopeptide repeat, ankyrin repeat and coiled-coil containing 1	XM_024980437.1	60	GTCTCGCTGCCGAAGAAA	GGCCTTGGAAGCAAATTCT
*TFRC*	Transferrin receptor	NM_001206577.1	72	AATCCCAGCGGTCTCTTTCT	ATAGGTGTCCATGGGAGTGC
*TNIK*	TRAF2 and NCK interacting kinase	XM_002684922.5	66	GTGCTCCAATGGGGAGAAAT	CCCAGCCCATTATCTGATTG
*TP53*	Tumor protein p53	NM_174201.2	62	CCTCTCCACAGCCAAAGAAG	ACCCACGGATCTGAAGAGTG
*TTYH1*	Tweety family member 1	NM_001077015.1	78	GGCACTGCTACATTGTCGTG	TCCTTCCAGGGTGTCTTCAC
*YES1*	YES proto-oncogene 1, Src family tyrosine kinase	NM_001101060.1	79	TGGCTTAGCAAGGTTAATTGAAG	CAGGAGCTGTCCACTTGATTG

**Table 2:** Impacts of IVM temperature (38.5 or 41.0 °C) by hIVM on abundance (counts per million) of COC transcripts involved in progesterone production/signaling, cellular signaling, β-catenin complex, cell cycle and cumulus cell expansion: Direct impact of 41.0 °C on first 6 hIVM versus delayed impact occurring after 4 (10 hIVM) or 18 (24 hIVM) h of recovery.

Role ^1^	Transcript Gene Symbol	IVM Temp (°C)	0 hIVM		Direct Impact—41 °C				Delayed Impact—41 °C			First Affected hIVM	Pooled SEM	hIVM × IVM Temp *p*-Value
					2 hIVM	4 hIVM	6 hIVM		10 hIVM ^2^	24 hIVM ^2^				
Progesterone production and/or signaling	*FDX1*	38.5	31,014		36,906 ^B^	38,258 ^B^	29,145 ^D^		49,090 ^A^	30,548 ^CD^		2	1066	<0.0001
		41.0			31,061 ^CD^	31,948 ^CD^	35,837 ^B^		29,414 ^D^	32,457 ^C^			1046	
	*FDXR*	38.5	19,012		18,666 ^A^	18,829 ^A^	15,001 ^B^		9210 ^D^	8690 ^DE^		2	641	<0.0001
		41.0			15,887 ^B^	14,265 ^B^	11,882 ^C^		12,406 ^C^	7325 ^E^			629	
	*HSD3B7*	38.5	23,899		20,030 ^DE^	16,954 ^EF^	22,002 ^CD^		13,857 ^F^	25,145 ^AB^		2	1105	<0.0001
		41.0			23,778 ^ABC^	22,299 ^BCD^	14,800 ^F^		24,505 ^ABC^	26,541 ^A^			1084	
	*HSP90AB1*	38.5	19,277		25,502 ^B^	24,980 ^B^	19,445 ^DE^		31,211 ^A^	20,030 ^DE^		2	762	<0.0001
		41.0			20,473 ^DE^	21,218 ^CD^	22,607 ^C^		19,056 ^E^	20,944 ^CDE^			748	
	*HSP90B1*	38.5	23,708		36,256 ^B^	37,361 ^B^	28,990 ^CD^		47,348 ^A^	26,655 ^D^		2	1783	<0.0001
		41.0			29,643 ^CD^	32,889 ^BC^	34,825 ^B^		26,638 ^D^	27,526 ^D^			1750	
	*PGRMC2*	38.5	24,783		32,263 ^B^	24,023 ^DE^	24,079 ^DE^		39,159 ^A^	24,315 ^DE^		2	1110	<0.0001
		41.0			27,529 ^C^	26,625 ^CD^	28,392 ^C^		21,418 ^E^	23,603 ^DE^			1090	
														
Cellular signaling	*BDKRB1*	38.5	162		5884 ^D^	7973 ^D^	23,533 ^AB^		16,566 ^C^	13,999 ^C^		4	1404	<0.0001
		41.0			4763 ^D^	14,058 ^C^	20,479 ^B^		26,552 ^A^	14,269 ^C^			1384	
	*IL6R*	38.5	16,582		15,944 ^A^	10,993 ^BC^	14,016 ^AB^		5789 ^D^	5191 ^D^		6	1249	<0.0001
		41.0			15,492 ^A^	14,070 ^AB^	9792 ^C^		15,762 ^A^	4442 ^D^			1226	
	*MAPK3*	38.5	17,830		11,943 ^BC^	13,841 ^B^	10,709 ^BC^		6097 ^DE^	20,264 ^A^		6	1243	<0.0001
		41.0			9008 ^CD^	10,236 ^BC^	4823 ^E^		18,981 ^A^	18,133 ^A^			1220	
	*MMP9*	38.5	176		7606 ^D^	11,332 ^D^	19,787 ^BC^		29,568 ^A^	22,617 ^B^		4	1723	<0.0001
		41.0			8165 ^D^	19,109 ^BC^	23,086 ^B^		21,046 ^BC^	16,782 ^C^			1692	
	*PRKAA1*	38.5	37,760		35,582 ^C^	37,618 ^BC^	36,713 ^BC^		32,688 ^D^	38,875 ^AB^		6	1079	<0.0001
		41.0			35,700 ^C^	37,383 ^BC^	31,995 ^D^		38,953 ^AB^	41,258 ^A^			1060	
														
β-catenin complex	*ESR2*	38.5	21,355		18,069 ^CD^	13,559 ^EF^	18,130 ^CD^		10,365 ^F^	21,473 ^AB^		4	1245	<0.0001
		41.0			20,216 ^ABC^	22,468 ^A^	15,440 ^DE^		18,653 ^BC^	21,174 ^ABC^			1225	
	*SF1*	38.5	22,248		21,450 ^CD^	25,698 ^A^	20,685 ^D^		25,154 ^AB^	22,285 ^CD^		4	794	0.0002
		41.0			21,781 ^CD^	22,659 ^CD^	23,441 ^ABC^		20,859 ^D^	23,282 ^BC^			779	
	*TNIK*	38.5	8676		9361 ^C^	11,149 ^C^	22,815 ^AB^		25,642 ^A^	11,532 ^C^		4	1155	<0.0001
		41.0			8423 ^C^	20,683 ^B^	22,000 ^B^		23,513 ^AB^	11,112 ^C^			1133	
														
Cell cycle	*ARHGAP6*	38.5	18,331		14,194 ^AB^	11,134 ^C^	14,578 ^AB^		5488 ^E^	12,221 ^BC^		4	901	<0.0001
		41.0			14,097 ^AB^	15,863 ^A^	8271 ^D^		13,123 ^BC^	13,621 ^ABC^			884	
	*ARHGAP31*	38.5	20,552		14,009 ^B^	15,930 ^B^	19,949 ^A^		15,498 ^B^	20,399 ^A^		2	1168	<0.0001
		41.0			20,971 ^A^	21,512 ^A^	15,282 ^B^		20,582 ^A^	19,919 ^A^			1146	
	*BANP*	38.5	13,469		11,478 ^C^	7153 ^D^	12,495 ^BC^		12,693 ^BC^	13,982 ^AB^		4	782	0.001
		41.0			12,157 ^BC^	12,186 ^BC^	15,965 ^A^		11,912 ^BC^	13,264 ^BC^			767	
	*MCM6*	38.5	31,056		36,647 ^B^	37,376 ^B^	29,007 ^F^		48,505 ^A^	31,138 ^DEF^		2	975	<0.0001
		41.0			30,353 ^DEF^	31,894 ^DE^	35,428 ^BC^		29,870 ^EF^	32,884 ^CD^			957	
	*MDM2*	38.5	24,352		27,166 ^BC^	29,707 ^A^	24,297 ^D^		29,670 ^A^	24,385 ^D^		2	708	<0.0001
		41.0			24,483 ^D^	23,951 ^D^	27,708 ^AB^		23,573 ^D^	25,395 ^CD^			695	
	*NR5A1*	38.5	28,435		17,431 ^DE^	15,953 ^E^	22,877 ^CD^		9508 ^F^	30,282 ^AB^		2	2079	<0.0001
		41.0			27,667 ^BC^	23,627 ^C^	11,861 ^EF^		26,127 ^BC^	34,189 ^A^			2040	
	*PAK2*	38.5	25,343		31,463 ^B^	29,649 ^BC^	25,106 ^D^		42,966 ^A^	25,673 ^D^		2	1062	<0.0001
		41.0			25,848 ^D^	26,542 ^CD^	30,117 ^B^		24,334 ^D^	27,044 ^CD^			1042	
	*TP53*	38.5	23,042		20,643 ^BCD^	13,140 ^F^	18,920 ^CDE^		14,524 ^F^	23,761 ^AB^		4	1176	0.0006
		41.0			21,137 ^BCD^	18,268 ^DE^	16,406 ^EF^		21,726 ^ABC^	24,536 ^A^			1155	
														
Cumulus expansion	*FSHR*	38.5	24,828		28,364 ^B^	29,031 ^AB^	22,211 ^CDE^		31,375 ^A^	20,167 ^E^		2	969	<0.0001
		41.0			23,195 ^CD^	22,326 ^CDE^	24,716 ^C^		20,966 ^DE^	21,674 ^DE^			951	
	*INHBB*	38.5	21,649		12,845 ^D^	18,161 ^C^	25,061 ^AB^		11,789 ^D^	24,157 ^AB^		2	1282	<0.0001
		41.0			26,341 ^AB^	23,189 ^B^	13,551 ^D^		22,898 ^B^	27,003 ^A^			1258	
	*PRDX2*	38.5	19,085		22,069 ^BC^	17,356 ^E^	19,722 ^CDE^		36,038 ^A^	21,472 ^CD^		2	926	<0.0001
		41.0			19,294 ^DE^	18,524 ^E^	24,320 ^B^		21,495 ^CD^	21,108 ^CD^			909	

Within each transcript, letters ^A–F^ indicate counts per million differ within column (IVM temperature) and row (hIVM). ^1^ The possible general role of each transcript in cumulus–oocyte complexes. ^2^ 41.0 °C was limited to first 6 hIVM followed by 38.5 °C for 4 (10 hIVM) or 18 (24 hIVM) h.

**Table 3:** Impact of IVM temperature (38.5 or 41.0 °C) by hIVM on Src-Family Kinase transcript abundance (counts per million) in cumulus–oocyte complexes: Direct impact of 41.0 °C on first 6 hIVM versus delayed impact occurring after 4 (10h IVM) or 18 (24 hIVM) h of recovery.

hIVM First Impacted by 41 °C	Transcript Gene Symbol	IVM Temp (°C)	0 hIVM		Direct Impact—41 °C				Delayed Impact—41 °C			Pooled SEM	hIVM × IVM Temp *p*-Value
					2 hIVM	4 hIVM	6 hIVM		10 hIVM ^1^	24 hIVM ^1^			
2	*SRC*	38.5	8976		8945 ^CD^	6742 ^DE^	12,058 ^BC^		4491 ^E^	8489 ^CD^		1382	0.0001
		41.0			15,994 ^A^	14,250 ^AB^	7105 ^DE^		9584 ^CD^	8902 ^CD^		1356	
4	*FYN*	38.5	44,884		48,207 ^CD^	39,394 ^F^	48,992 ^CD^		70,059 ^A^	44,866 ^DE^		1787	<0.0001
		41.0			49,540 ^CD^	50,928 ^C^	57,706 ^B^		49,554 ^CD^	40,928 ^EF^		1754	
4	*LCK*	38.5	1777		2340 ^D^	3652 ^C^	5652 ^AB^		6157 ^A^	4542 ^BC^		490	0.0008
		41.0			1835 ^D^	5537 ^AB^	5726 ^AB^		4090 ^C^	3616 ^C^		483	
6	*FGR*	38.5	3498		5199 ^AB^	3369 ^DEF^	6109 ^A^		2597 ^F^	2712 ^EF^		462	0.005
		41.0			5021 ^ABC^	3920 ^CDE^	4388 ^BCD^		4385 ^BCD^	2526 ^F^		454	
10	*LYN*	38.5	4529		4079 ^B^	3676 ^BC^	6067 ^A^		1877 ^C^	4881 ^AB^		748	0.0008
		41.0			4165 ^AB^	5155 ^AB^	4515 ^AB^		6120 ^A^	3697 ^BC^		735	
10	*YES1*	38.5	17,446		20,015 ^B^	15,985 ^D^	17,959 ^BCD^		23,810 ^A^	19,558 ^BC^		902	0.02
		41.0			19,081 ^BC^	17,146 ^CD^	20,443 ^B^		20,456 ^B^	19,798 ^B^		885	

Within each transcript, letters ^A–F^ indicate counts per million differ within column (IVM temperature) and row (hIVM). ^1^ 41.0 °C was limited to first 6 hIVM followed by 38.5 °C for 4 (10 hIVM) or 18 (24 hIVM) h.

**Table 4:** Impacts of IVM temperature (38.5 or 41.0 °C) by hIVM on abundance (counts per million) of COC transcripts found to be significantly affected by temperature in cumulus, oocyte, and/or follicular fluid in other published studies: Direct impact of 41.0 °C on first 6 hIVM versus delayed impact occurring after 4 (10 hIVM) or 18 (24 hIVM) h of recovery.

Transcript Gene Symbol	IVM Temp (°C)	0 hIVM		Direct Impact—41 °C				Delayed Impact—41 °C			First hIVM Affected by IVM Temp	Pooled SEM	hIVM × IVM Temp *p*-Value
				2 hIVM	4 hIVM	6 hIVM		10 hIVM ^1^	24 hIVM ^1^				
*CCDC25*	38.5	31,308		33,850 ^D^	40,611 ^B^	33,038 ^DE^		49,178 ^A^	32,556 ^DEF^		2	1064	<0.0001
	41.0			29,014 ^G^	32,379 ^DEF^	37,339 ^C^		30,641 ^EFG^	29,749 ^FG^			1044	
*CTSB*	38.5	25,243		22,336 ^B^	12,916 ^C^	24,597 ^AB^		27,109 ^A^	26,467 ^A^		4	1368	<0.0001
	41.0			25,336 ^AB^	26,263 ^A^	27,537 ^A^		24,296 ^AB^	26,826 ^A^			1342	
*ETFA*	38.5	26,445		29,886 ^BC^	34,166 ^A^	27,454 ^CD^		30,806 ^B^	27,216 ^CD^		2	1004	0.01
	41.0			26,773 ^D^	26,835 ^D^	27,252 ^CD^		26,340 ^D^	25,133 ^D^			985	
*HTATIP2*	38.5	22,469		22,176 ^AB^	12,638 ^E^	18,476 ^CD^		17,754 ^CD^	23,739 ^A^		4	971	0.0002
	41.0			19,850 ^BC^	18,717 ^CD^	16,999 ^D^		18,541 ^CD^	22,116 ^AB^			954	
*PEAK1*	38.5	18,058		13,853 ^E^	25,209 ^A^	17,703 ^C^		11,974 ^E^	20,053 ^BC^		4	1253	<0.0001
	41.0			14,560 ^DE^	17,381 ^CD^	12,373 ^E^		19,041 ^C^	23,167 ^AB^			1233	
*RBM3*	38.5	32,657		40,004 ^B^	40,142 ^B^	31,047 ^C^		51,722 ^A^	31,677 ^C^		2	1337	<0.0001
	41.0			31,926 ^C^	34,069 ^C^	37,955 ^B^		31,554 ^C^	33,707 ^C^			1312	
*SCN7A*	38.5	340		1000 ^C^	2702 ^B^	4398 ^A^		1787 ^BC^	1012 ^C^		4	416	0.03
	41.0			1136 ^C^	4925 ^A^	4334 ^A^		2864 ^B^	1098 ^C^			408	
*SLC26A2*	38.5	26,849		24,262 ^B^	19,633 ^C^	27,371 ^A^		15,778 ^D^	28,389 ^A^		2	913	<0.0001
	41.0			29,649 ^A^	27,540 ^A^	24,940 ^B^		28,017 ^A^	28,961 ^A^			898	
*SNX22*	38.5	20,829		16,065 ^AB^	13,351 ^BC^	18,141 ^A^		8793 ^D^	13,042 ^C^		6	1138	<0.0001
	41.0			14,610 ^BC^	15,116 ^BC^	13,859 ^BC^		18,427 ^A^	13,647 ^BC^			1118	
*TANC1*	38.5	15,236		15,258 ^AB^	8533 ^CD^	9930 ^C^		6228 ^D^	13,946 ^B^		6	1005	<0.0001
	41.0			15,930 ^AB^	10,257 ^C^	5684 ^D^		18,056 ^A^	16,462 ^AB^			986	
*TTYH1*	38.5	27,976		27,610 ^B^	30,916 ^A^	24,524 ^CDEF^		22,724 ^F^	22,895 ^EF^		4	876	0.0005
	41.0			26,493 ^BC^	25,236 ^BCDE^	26,101 ^BCD^		23,859 ^DEF^	24,693 ^CDEF^			860	

Within each transcript, letters ^A–G^ indicate counts per million differ within column (IVM temperature) and row (hIVM). ^1^ Possible general role of each transcript in cumulus–oocyte complexes. 41.0 °C was limited to first 6 hIVM followed by 38.5 °C for 4 (10 hIVM) or 18 (24 hIVM) h.

**Table 5:** The earliest time period when 41 °C exposure for a maximum of 6 h first impacted the abundance of 43 different transcripts.

hIVM	Transcripts First Impacted by Exposure to 41 °C (#) ^1^	Transcripts of Higher Abundance (#)	Transcripts of Lower Abundance (#)	Cumulative Proportion Affected (%) ^2^	Transcripts (Higher or Lower Abundance) ^3^
2	19	6	13	19/43 (44.1)	***ARHGAP31* ↑**, *CCDC25* ↓, ***ETFA* ↓**, *FDX1* ↓, *FDXR* ↓, *FSHR* ↓, *HSD3B7* ↑, *HSP90AB1* ↓, *HSP90B1* ↓, *INHBB* ↑, *MDM2* ↓, ***MCM6* ↓**, *NR5A1* ↑, ***PAK2* ↓**, *PGRMC2* ↓, ***PRDX2* ↓**, ***RBM3* ↓**, ***SLC26A2* ↑**, *SRC* ↑
4	15	12	3	34/43 (79.1)	***ARHGAP6* ↑**, ***BANP* ↑**, *BDKRB1* ↑, *CTSB* ↑, *ESR2* ↑, ***FYN* ↑**, ***HTATIP2* ↑**, *LCK* ↑, *MMP9* ↑, ***PEAK1* ↓**, ***SCN7A* ↑**, *SF1* ↓, ***TNIK* ↑**, *TP53* ↑, ***TTYH1* ↓**
6	6	0	6	40/43 (93.0)	*FGR* ↓, *IL6R* ↓, *MAPK3* ↓, *PRKAA1* ↓, *SNX22* ↓, ***TANC1* ↓**
10	3	2	1	43/43 (100)	***CAV1* ↑ **^4^, *LYN* ↑, *YES1* ↓
24	0	0	0	-	None

^1^ The earliest—but not necessarily the only—hour of in vitro maturation (hIVM) in which transcript abundance were significantly different (hIVM × IVM temperature; *p* < 0.05), ^2^ The proportion of transcripts affected at the indicated hIVM or earlier, ^3^ ↑ = higher abundance, ↓ = lower abundance, ^4^ *CAV1* data were analyzed only including 2, 4, 6, and 10 hIVM. Orange colored text: cumulus derived transcripts from Klabnik et al. [23].

## Data Availability

Data presented in this study may be available for viewing upon submission of a reasonable request from the co-corresponding author at jlk0066@auburn.edu.

## Abbreviations

The following abbreviations are used in this manuscript:

*AMPK*	AMP-activated protein kinase
*ARHGAP31*	Rho GTPase activating protein 31
*ARHGAP6*	Rho GTPase activating protein 6
*BANP*	BTG3 associated nuclear protein
*BDKRB1*	Bradykinin receptor B1
*CAV1*	Caveolin 1
*CCDC25*	Coiled-coil domain containing 25
*CCDC80*	Coiled-coil domain containing 80
*COC*	Cumulus–oocyte complex
*CPM*	Counts per million
*CTSB*	Cathepsin B
*DEGs*	Differentially expressed genes
*ESR2*	Estrogen receptor 2
*ERK*	Extracellular signal-regulated kinase
*ETFA*	Electron transfer flavoprotein subunit alpha
*FDX1*	Ferredoxin 1
*FDXR*	Ferredoxin reductase
*FGR*	FGR proto-oncogene, Src family tyrosine kinase
*FSHR*	Follicle stimulating hormone receptor
*FYN*	FYN proto-oncogene, Src family tyrosine kinase
*GVBD*	Germinal vesicle breakdown
*h*	Hour(s)
*HEAT*	Higher estrus-associated temperature
*hIVM*	Hours of in vitro maturation
*HS*	Heat shock
*HSD3B7*	Hydroxy-delta-5-steroid dehydrogenase, 3-beta- and steroid delta-isomerase 7
*HSP90AB1*	Heat shock protein 90 alpha family class B member 1
*HSP90B1*	Heat shock protein 90 beta family member 1
*HTATIP2*	HIV-1 Tat interactive protein 2
*IL6R*	Interleukin 6 receptor
*INHBB*	Inhibin subunit beta B
*LCK*	LCK proto-oncogene, Src family tyrosine kinase
*LH*	Luetinizing hormone
*LYN*	LYN proto-oncogene, Src family tyrosine kinase
*MAPK3*	Mitogen-activated protein kinase 3
*MCM6*	Minichromosome maintenance complex component 6
*MDM2*	MDM2 proto-oncogene
*MMP9*	Matrix metallopeptidase 9
*NR5A1*	Nuclear receptor subfamily 5 group A member 1
*PAK2*	p21 (RAC1) activated kinase 2
*PEAK1*	Pseudopodium enriched atypical kinase 1
*PGRMC2*	Progesterone receptor membrane component 2
*PRDX2*	Peroxiredoxin 2
*PRKAA1*	Protein kinase AMP-activated catalytic subunit alpha 1
*RAI14*	Retinoic acid induced 14
*RBM3*	RNA binding motif protein 3
*SCN7A*	Sodium voltage-gated channel alpha subunit 7
*SF1*	Splicing factor 1
*SLC26A2*	Solute carrier family 26 member 2
*SNAP91*	Synaptosome associated protein 91
*SNX22*	Sorting nexin 22
*SRC*	SRC proto-oncogene, non-receptor tyrosine kinase
*TANC1*	Tetratricopeptide repeat, ankyrin repeat and coiled-coil containing 1
*TFRC*	Transferrin receptor
*TN*	Thermoneutral
*TNIK*	TRAF2 and NCK interacting kinase
*TP53*	Tumor protein p53
*TTYH1*	Tweety family member 1
*YES1*	YES proto-oncogene 1, Src family tyrosine kinase
